# Quality of platelet concentrates after three‐day storage following 265 nm ultraviolet C‐light‐emitting diode irradiation

**DOI:** 10.1111/tme.70025

**Published:** 2025-10-09

**Authors:** Tomoya Hayashi, Yoshihiko Sakurai, Yoshihiro Fujimura, Kumiko Oguma, Yuichi Mishima, Fumiya Hirayama, Yoshihiko Tani, Yoshihiro Takihara, Takafumi Kimura

**Affiliations:** ^1^ Department of Research and Development, Japanese Red Cross Kinki Block Blood Center Japanese Red Cross Society Osaka Ibaraki Japan; ^2^ Japanese Red Cross Nara Blood Center Nara Japan; ^3^ Japanese Red Cross Osaka Blood Center Osaka Japan; ^4^ Department of Urban Engineering The University of Tokyo Tokyo Japan; ^5^ Japanese Red Cross Society Central Blood Institute Tokyo Japan

**Keywords:** 265 nm ultraviolet, LED, platelet concentrate, quality assessment, storage

## Abstract

**Objectives:**

We aimed to investigate the quality of platelets after storage following irradiation with ultraviolet C (UVC) light‐emitting diodes (LED).

**Background:**

Controlling transfusion‐related infections, particularly bacterial contamination of platelet concentrates (PCs), is urgently required. UVC‐LEDs have attracted considerable attention as potential solutions to this problem.

**Methods:**

PCs (5.5 mL) were irradiated with 265 nm UVC‐LED for up to 60 min and then stored at 22°C with shaking for 3 days. PC quality parameters, such as platelet count, biochemical profiles, including electrolytes and metabolism, activation markers and platelet aggregability, were analysed before and after storage. Prior to the storage study, to validate the appropriateness of the UVC‐LED dose used, the PCs were inoculated with *Staphylococcus aureus* or *Bacillus cereus*, and their colony‐forming ability was evaluated after irradiation with the same dose of UVC‐LED.

**Results:**

We confirmed that *S. aureus* and *B. cereus* colonies decreased with the irradiation dose (by 1.7 log and 1.2 log at 38.7 and 40.4 mJ cm^−2^, respectively). Platelet count decreased immediately after 60‐minute irradiation to 32.5 mJ cm^−2^, reaching approximately 80% of the level in the control without irradiation, but no further decrease was recorded after storage. The biochemical profiles and activation markers showed little alterations.

**Conclusion:**

These results indicate that UVC‐irradiated platelets maintain sufficient quality for practical use even after storage. Although this was a bench‐scale study, our findings suggest that irradiation of PCs with 265 nm UVC‐LED may enhance the safety of blood transfusions while preserving their efficacy.

## 1 INTRODUCTION

Reducing the levels of infectious pathogens in blood products remains a critical issue in transfusion medicine. In particular, preventing bacterial contamination of platelet concentrates (PCs), which are usually stored at room temperature, remains an urgent concern.

Conventionally, controlling bacterial infections in PCs relies on three main approaches. First, culture‐based methods, such as the BacT/ALERT system (bioMérieux, Marcy‐l'Étoile, France),^1^, ^2^ extend the shelf‐life (i.e., delayed expiration date) of PCs. Second, storing PCs at low temperatures to suppress bacterial growth allows for shelf lives of 14–21 days.^3^ However, such products are restricted for use in traumatic haemorrhage and are unsuitable for prophylactic transfusion because of their reduced biological lifespan. Third, pathogen reduction technologies (PRTs), such as the INTERCEPT (using amotosalen) and Mirasol methods (using vitamin B_2_), have been applied to PCs.^4^, ^5^, ^6^, ^7^ The INTERCEPT method requires an additional adsorption step to remove amotosalen and its photoproducts.^6^ The recently developed THERAFLEX UV‐Platelet method uses 254 nm ultraviolet C (UVC) irradiation from a mercury (Hg) lamp and does not require any photosensitizers.^8^, ^9^ Furthermore, UVC irradiation alone is effective for pathogen inactivation in blood products and eliminates the need for photosensitizers, thereby reducing contamination risks and processing time.^10^, ^11^ Recently, non‐inferiority has been reported in clinical trials.^12^ However, because UVC treatment may affect platelet function, the quality and stability of PCs during storage depend on the PRT employed.^13^

As an alternative to Hg lamps, we recently applied 265 nm UVC‐light‐emitting diode (LED) irradiation for pathogen reduction and obtained promising results for PC formulations at the laboratory scale.^14^ UVC‐LEDs offer several advantages; they are environmentally friendly, energy‐efficient, long‐lasting and highly durable. However, in our previous study, we did not assess the quality of UVC‐irradiated PCs after storage. Except for one study that used 254 nm UVC,^15^ few investigations have examined the post‐storage quality of UVC‐irradiated PCs, and none have employed 265 nm UVC.

Therefore, the aim of the present study was to evaluate the quality of PCs after 265 nm UVC‐LED irradiation and subsequent storage. Specifically, the platelet count, pH, biochemical parameters, activation markers and platelet aggregability were assessed in PCs stored for 3 days after irradiation.

## 2 MATERIALS AND METHODS

### 2.1 Ethical consideration

Written informed consent was obtained from randomly selected volunteer blood donors before sample collection. All procedures were conducted in accordance with the Ethical Guidelines for Medical and Health Research Involving Human Subjects issued by the Ministry of Health, Labour and Welfare of Japan. The study protocol was approved by the Ethics Committee of the Japanese Red Cross Blood Institute (approval number: 2021–006).

### 2.2 UVC‐LED irradiation

PCs were collected by apheresis using a Trima accel (Terumo BCT, Tokyo, Japan) or Haemonetics Component Collection System (CCS, Haemonetics Japan, Tokyo, Japan), and stored at 22°C with agitation until use. The day of collection was designated as day 1. On day 3, 5.5 mL of PCs was placed in a quartz glass Petri dish (inner diameter: 28 mm, depth: 15 mm; As One, Osaka, Japan), achieving a sample depth of 1.0 cm. The dish was covered with a GE214 glass lid (Asahi Glass, Tokyo, Japan) and irradiated using a bench‐scale UVC‐LED setup as previously described^14^ The setup in this study is described in Supporting Information (Figure S1). The UVC‐LEDs were operated at a constant direct current of 51.5 mA per unit, generating an incident irradiance of 1.3 mW cm^−2^ at the surface of the PCs, as measured using a spectral radiometer (USHIO, Tokyo, Japan). The incident irradiance was corrected using the Beer–Lambert Law, following the method reported by Bolton and Linden.^16^ To calculate the amount of UVC‐LED light exposure, 10 μL aliquots of PCs were diluted in the ratio 1:100 with 990 μL of platelet additive solution type E (PAS‐E), and the absorbance at 265 nm was measured. The average irradiance was calculated as previously described,^14^, ^16^, ^17^ and the average fluence was determined as the product of the average irradiance and exposure time. Under these conditions, the transmission rate of the GE214 glass lid was calculated to be 90%.^18^

### 2.3 Bacterial inoculation of PCs

To confirm the effectiveness of the UVC‐LED irradiation dose used in the study, we evaluated its bactericidal activity against *Staphylococcus aureus* (NBRC 3060) and *Bacillus cereus* (NBRC 3001), both provided by the NITE Biological Resource Center (Tokyo, Japan). Bacteria were cultured on agar plates overnight at 30°C. A single colony was then selected and diluted with day 3 PCs to a final concentration (f.c.) of 500 colony‐forming units (CFU) mL^−1^. For both irradiated and non‐irradiated inoculated PCs, bacterial CFU counts were measured at predetermined time points as previously described.^14^

### 2.4 Storage of UVC‐LED irradiated PCs

For the storage experiment, aliquots (approximately 5 mL) of each sample (with or without UVC‐LED irradiation) were transferred to 15 mL NIPRO Culture Bags (NIPRO, Osaka, Japan) made of polyolefins with high oxygen permeability^19^ at 0, 20, 40 and 60 min after the start of the experiment. The bags containing PCs were stored at 22°C on a flat‐bed rotator set to 50–60 cycles per minute for 3 days. Evaluations were performed on day 3 (immediately after UVC‐LED irradiation) and day 6 (3 days after storage), with the exception of some tests, such as the platelet aggregation test and hypotonic shock response test, which were carried out only on day 6. UVC‐LED irradiation experiments were conducted using eight different PC products. Details regarding the selection of platelet storage bags used in this study are provided in Supporting Information (Selection of PC storage bags), including Supporting Information (Figure S2) and Supporting Information (Figure S3).

## 3 SUBJECTS STUDIED

### 3.1 Visual inspection and evaluation of platelet count and biochemical profiles

We evaluated PC parameters before storage (immediately after UVC‐LED irradiation) and 3 days after storage. Swirling and the presence of platelet aggregates were assessed by visual inspection. Platelet counts were measured using the XS‐800i cell counter (Sysmex, Tokyo, Japan). Blood gas parameters (pO_2_ and pCO_2_), pH, electrolytes (Na^+^ and K^+^), and metabolic markers including glucose and lactate were measured using a GEM 5000 blood gas analyser (Welfen‐IL Japan, Tokyo, Japan).

### 3.2 Platelet activation markers

Platelet activation by UVC‐LED irradiation was investigated using platelet activation markers, such as PAC‐1, CD62P and Annexin V. PAC‐1 is an antibody that recognizes activated and structurally altered GPIIb/GPIIIa on platelets. CD62P (P‐selectin) is a component of the platelet α‐granule membrane and is expressed on the activated platelet surface^20^, along with the release of fibrinogen, VWF, PF4, β‐TG, etc. Annexin V is a protein that has an affinity for phospholipids, such as phosphatidylserine, and binds to phospholipids expressed on the surface of activated platelets. Details of the method used to evaluate platelet activation are provided in the Supporting Material (Platelet activation markers).

### 3.3 Agonist‐induced platelet aggregation

Agonist‐induced platelet aggregation was assessed using a Hematoracer 912 (DS Medical, Tokyo, Japan). Platelet‐rich plasma (PRP) was prepared by adding AB‐type normal plasma to the sample PCs and adjusting the final platelet concentration to 200 × 10^3^ μL^−1^. Adenosine diphosphate (ADP; f.c. 10 μM; Sigma Aldrich Japan, Tokyo, Japan), collagen (f.c. 2.5 μg mL^−1^; Collagen Reagent Horm, Moriya Sangyo, Tokyo, Japan), and a combination of ADP (f.c. 5 μM) and collagen (f.c. 2.5 μg mL^−1^) were used as agonists. The maximum transmittance was recorded as a measure of platelet aggregability.

### 3.4 Hypotonic shock response (HSR)

The ability of platelets to respond to a hypotonic environment was determined as HSR using the standard method described by Holme and colleagues.^21^, ^22^ Platelet aliquots stored for three days after UVC irradiation were diluted with plasma at a final platelet concentration of 200 × 10^3^ μL^−1^. A 100% transmittance reference at 610 nm was established using a cuvette containing 400 μL of plasma and 200 μL of physiological saline. Another aliquot (400 μL) of the platelet sample was mixed with 200 μL of distilled water in a cuvette, and the percent transmittance (%*T*) was monitored for 6 min. The peak %*T* value was defined as T1, and the %T exactly 5 min after the peak was defined as T2. Additionally, 400 μL of the platelet sample was mixed with 200 μL of physiological saline, and the %*T* at the time point corresponding to T2 was defined as T0. The percentage of HSR is calculated using the following formula:

(T1 – T2) / (T1 – T0) × 100.

### 3.5 Statistical analysis

Statistical differences between samples were evaluated using the Bonferroni multiple comparison test and/or a paired *t*‐test when no significant difference in variance was observed based on the F‐test. P‐values were calculated using GraphPad Prism 8 (GraphPad Software, San Diego, CA, USA), and *p* < 0.05 was considered statistically significant.

## 4 RESULTS

### 4.1 UVC‐LED irradiation

In PCs diluted, in a ratio 1:100, with PAS‐E, the absorbance at 265 nm ranged from 0.522 to 0.645 (*n* = 8) as summarized in Supporting Information (Table S1), and correlated well with the platelet count (*r* ^2^ = 0.507) as shown in Supporting Information (Figure S4), which was in good accordance with our previous findings.^14^ The average fluences provided by irradiation for 20, 40 and 60 min were 10.8 ± 0.8 (range, 9.6–11.9), 21.7 ± 1.6 (19.3–23.8), and 32.5 ± 2.4 (28.9–35.7) mJ cm^−2^, respectively.

### 4.2 Bacterial count reduction in PCs during irradiation with UVC‐LED

The survival rates of *S. aureus* and *B. cereus* decreased in a UVC‐LED irradiation‐time‐dependent manner. The fluence required for consistent bacterial reduction was lower for *S. aureus* than for *B. cereus*, as provided in Supporting Information (Figure S5A) and Supporting Information (Figure S5B), respectively. The fluences required to achieve a 1‐log reduction were 22.5 mJ cm^−2^ for *S. aureus* and 33.3 mJ cm^−2^ for B. *cereus*, as shown in Supporting Information (Figure S5C) and Supporting Information (Figure S5D), respectively. Sixty minutes of irradiation significantly reduced the viable bacterial counts to near the detection limit of the assay, indicating that the applied dose was sufficient to achieve effective bacterial inactivation.

### 4.3 PC quality after storage following UVC‐LED irradiation

#### 4.3.1 Visual inspections

All PC samples exhibited swirling both before (immediately after UVC irradiation, day 3) and after the three‐day storage period (day 6). However, a time‐dependent increase in the number of small aggregates was observed before storage, which is consistent with our previous findings,^14^ and during the post‐storage visual evaluation (data not shown).

#### 4.3.2 Platelet count

**FIGURE 1:**
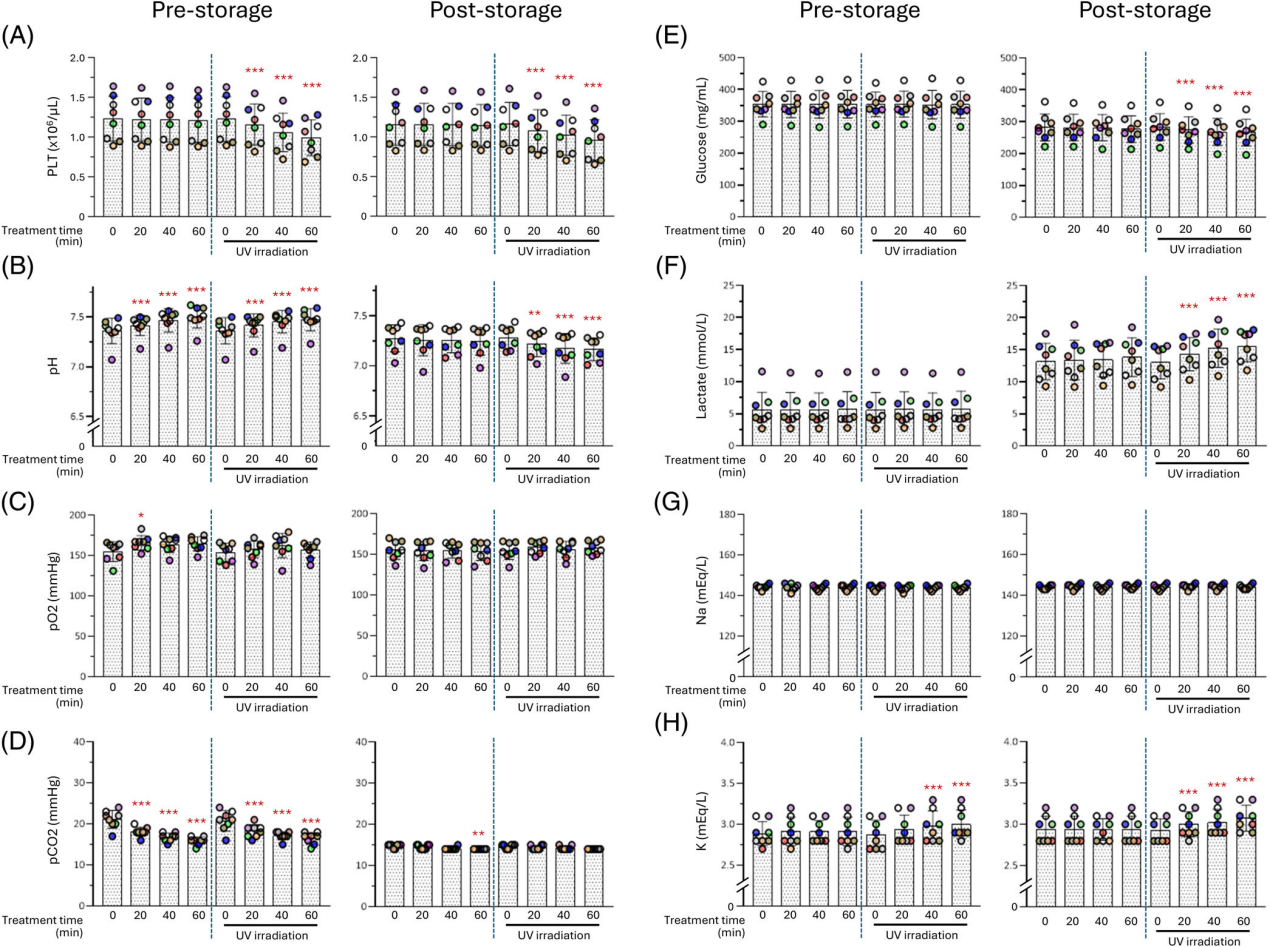
Quality of platelet concentrates (PCs) with or without 265 nm UVC‐LED irradiation before and after a three‐day storage period (I). The evaluation was conducted immediately after irradiation (day 3; pre‐storage, left) and 3 days after storage (day 6; post‐storage, right) as described Subject Studied section. (A) PLT, platelet count: (B) pH: (C) pO_2_: (D) pO_2_: (E) Glucose concentrate: (F) Lactate concentrate: (G) Na ion concentrate: (H) Na ion concentrate. *p < 0.05, **p < 0.01, ***p < 0.005. The *p*‐values represent comparisons with time 0 under each condition, with or without irradiation.

Immediately after irradiation, the platelet count decreased significantly in a time‐dependent manner. After 60‐minute irradiation, the count had decreased to approximately 80% of that of non‐irradiated PCs (Figure 1A, left). However, in 60‐minute irradiated PCs, the platelet count after the three‐day storage period showed no significant alteration compared to that before storage (*p* = 0.290) (Figure 1A, right).

#### 4.3.3 Biochemical profiles

During the 60‐minute irradiation treatment, pCO_2_ declined in a time‐dependent manner under both irradiated and non‐irradiated conditions (Figure 1D, left). Correspondingly, a time‐dependent increase in the pH was observed (Figure 1B, left). After the three‐day storage period, the pCO_2_ levels decreased to levels similar to those in non‐irradiated PCs (Figure 1D, right) and the pH was significantly reduced in a time‐dependent manner (Figure 1B, right). pO_2_, glucose and lactate levels showed limited differences between irradiated and non‐irradiated conditions even after the 60‐minute treatment (Figure 1C,E,F; left). In contrast, after the three‐day storage period, glucose levels significantly decreased (Figure 1E, right), whereas lactate levels increased significantly under irradiated conditions (Figure 1F, right). For electrolytes, K^+^ levels increased in an irradiation time‐dependent manner immediately after irradiation (Figure 1H, left), and a similar trend was observed after the three‐day storage period (Figure 1H, right). In contrast, the Na^+^ levels were not significantly different before and after storage with or without irradiation (Figure 1G).

#### 4.3.4 Platelet activation markers

**FIGURE 2:**
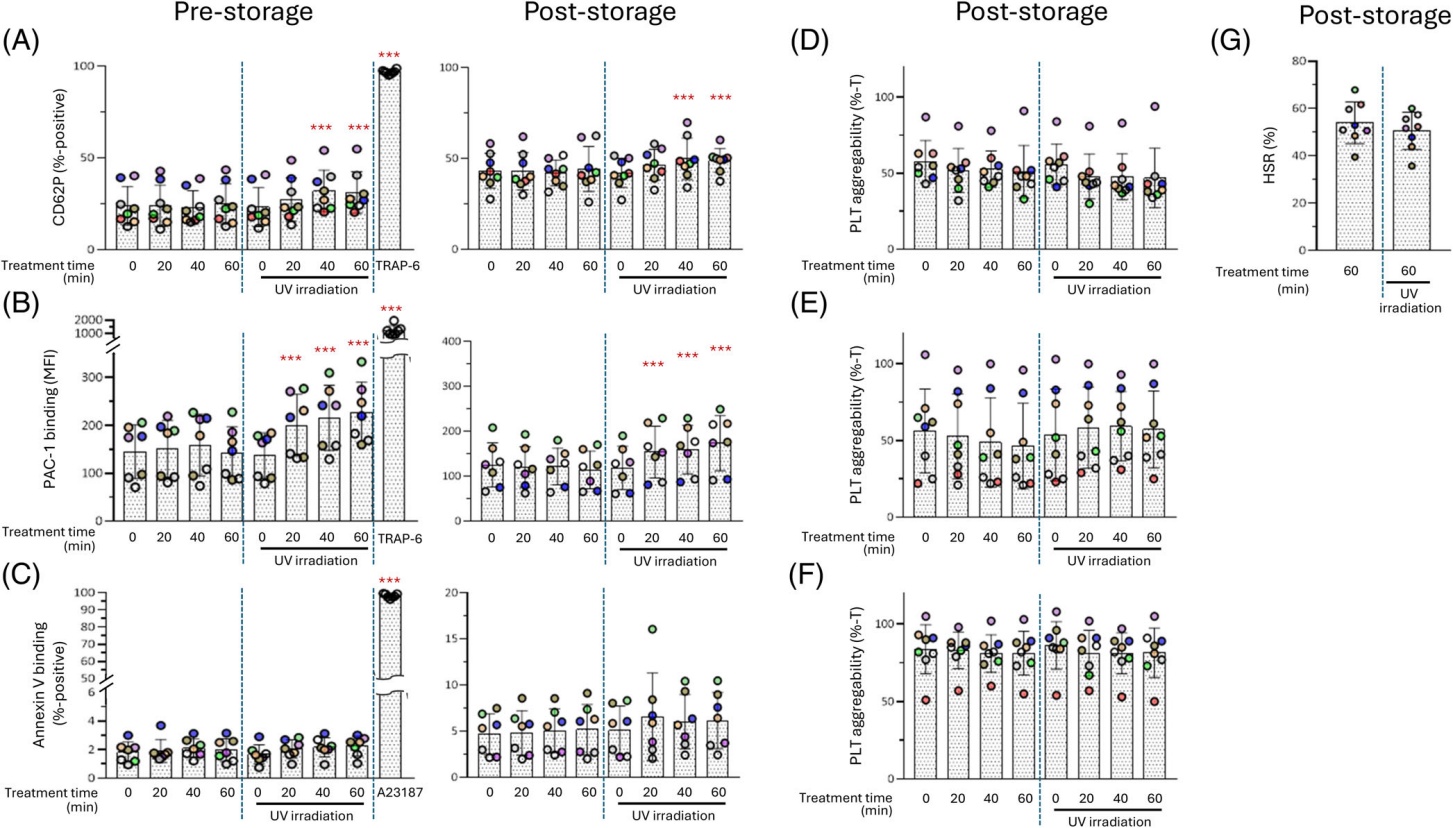
Quality of platelet concentrates (PCs) with or without 265 nm UVC‐LED irradiation before and after a three‐day storage period (II). The evaluation was conducted immediately after irradiation (day 3; pre‐storage, A left, B left, C left) and 3 days after storage (day 6; post‐storage, A right, B right, C right, D, E, F) as described Subject Studied section. (A) CD62p expression on platelet suraface: (B) PAC‐1 binding: (C) Annexin V binding: (D) platelet aggregability induced ADP (final concentration 10 μM): (E) platelet aggregability induced by collagen (final concentration 2.5 μg mL^−1^): (F) platelet aggregability induced by combination ADP (final concentration 5 μM) and collagen(final concentration 2.5 μg mL^−1^): (G) HSR, hypotonic shock response. Note that the vertical axis scales differ between the left and right panels in B and C. Independent experiments were performed using TRAP‐6 in A and C and A23187 in B as positive controls. *p < 0.05, **p < 0.01, ***p < 0.005. The *p*‐values represent comparisons with time 0 under each condition, with or without irradiation.

Immediately after irradiation, CD62P expression tended to increase with longer irradiation times (40 and 60 min) (Figure 2A, left). After the three‐day storage period, a similar trend was observed (Figure 2A, right). In addition, Annexin V binding showed no notable change immediately after irradiation, and no significant difference was observed after storage compared to non‐irradiated PCs (Figure 2C). PAC‐1 binding significantly increased immediately after irradiation (Figure 2B, left) and after storage (Figure 2B, right), in an irradiation time‐dependent manner.

### 4.4 Platelet aggregability after storage

**FIGURE 3:**
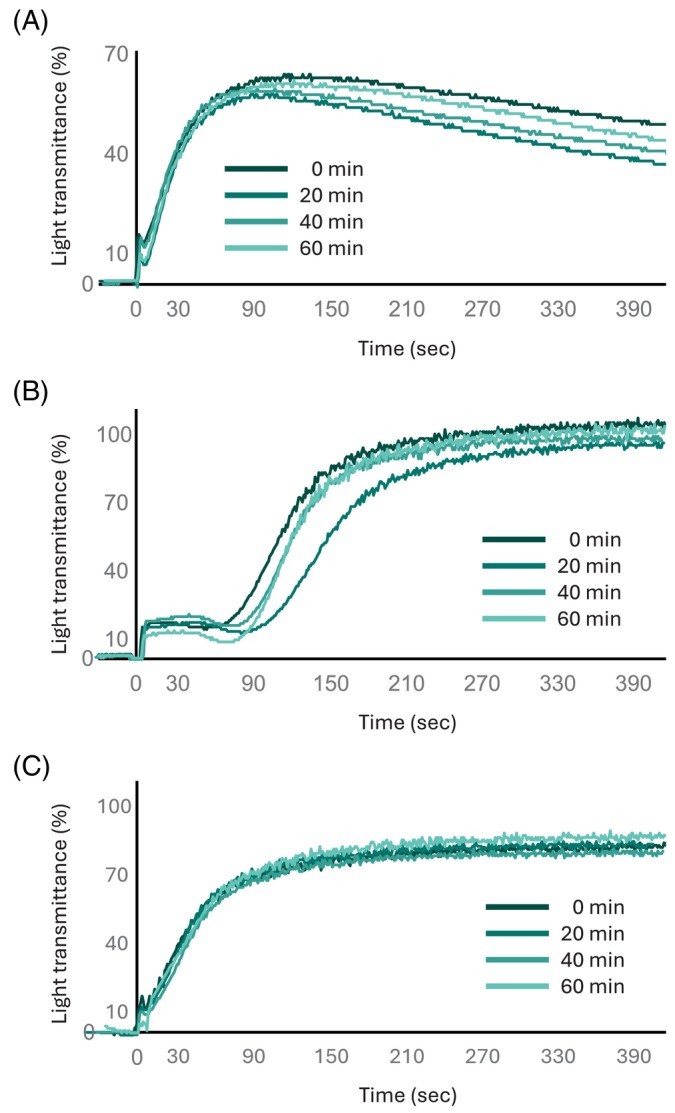
Platelet aggregation in UVC‐LED–irradiated PCs. Representative aggregation traces: ADP (A), collagen (B) and ADP+ collagen (C) as agonists. PCs irradiated with UVC for 0, 20, 40 and 60 min were stored for 3 days as described in the Materials and Methods section. Then, PRPs were prepared and applied for agonist‐induced platelet aggregation analysis as described in the Subject Studied section.

In ADP‐induced aggregation after the three‐day storage period, irradiated PCs exhibited a maximum aggregation response of approximately 80% compared with non‐irradiated PCs, although the difference was not statistically significant (Figures 2D and 3A). For collagen‐induced aggregation, the maximum aggregability of irradiated and non‐irradiated PCs was comparable (Figures 2E and 3B). In ADP plus collagen‐induced aggregation, no significant difference in maximum aggregation was observed regardless of the irradiation dose, and the aggregation curves were nearly identical (Figures 2F and 3C).

### 4.5 HSR after storage

After the three‐day storage period, no significant difference in the HSR was observed between irradiated and non‐irradiated PCs (Figure 2G).

## 5 DISCUSSION

Although our previous study demonstrated the bactericidal effects of UVC‐LED irradiation,^14^ it did not address the quality of irradiated PCs after storage. In the present study, we showed that platelet function was preserved, suggesting that UVC‐LED irradiation may retain PC quality even after storage.

Prior to conducting the storage experiments, we confirmed the effectiveness of the irradiation dose used in this study by evaluating its bactericidal activity against two bacterial species and then proceeded to investigate post‐storage platelet quality.

First, in platelets before storage, that is, immediately after UVC irradiation, a decrease in platelet count was observed in an irradiation time–dependent manner, which may have been caused by platelet aggregation and/or lysis.

During irradiation, it is likely that an adequate oxygen supply was maintained as the pO_2_ levels remained unchanged. In contrast, the pCO_2_ levels decreased over time, with or without irradiation, possibly because of the uniform distribution of CO_2_ in the plasma by magnetic stirring. This may have allowed high‐concentration CO_2_ to move toward the gas–liquid interface and volatilise, thereby increasing the pH. For electrolytes, increased irradiation time was associated with elevated K^+^, suggesting potassium leakage from platelets. This indicates potential membrane instability induced by UVC irradiation, which could contribute to platelet lysis.

In contrast, small platelet aggregates were observed, as previously reported.^14^ Platelets are activated upon exposure to 254 nm UVC irradiation,^13^, ^15^ and small platelet aggregates may be formed due to platelet activation. However, we previously reported that 265 nm UVC‐LED irradiation did not induce platelet activation based on PAC‐1 binding and CD62P expression analyses.^14^ In the present study, when the irradiation time was extended to 60 min, activation markers were further evaluated. These included CD62P, an adhesion molecule expressed upon platelet activation, especially granule release; PAC‐1, which binds to GPIIb/IIIa on activated platelets; and Annexin V, which binds to PS exposed on the surface of activated platelets. Significant changes in CD62P expression and PAC‐1 binding were observed immediately after irradiation. After storage, the levels of both activation markers were higher in irradiated PCs than in non‐irradiated PCs. Nonetheless, the increase was much lower than that in the TRAP‐6–stimulated positive controls, suggesting that it was not clinically significant. No alterations were observed in Annexin V binding, regardless of the presence or absence of irradiation, or before or after storage. These findings suggest that a slight degree of platelet lysis and/or aggregation due to weak platelet activation may have contributed to the observed reduction in the platelet count. However, neither of these effects appeared to have a substantial impact on platelet aggregability.

Notably, no further loss of platelets was observed during storage after irradiation. After storage, a decrease in glucose concentration, reflecting increased glucose consumption and an increase in lactate levels were observed under irradiation, suggesting that platelet metabolic function was affected. These findings are consistent with the slight increase in platelet metabolism previously reported in a study using 254 nm UVC irradiation.^15^ However, the blood gas parameters after the three‐day storage period were similar to those of the non‐irradiated controls. Although pH levels decreased significantly in an irradiation dose–dependent manner after storage, the magnitude of change (7.28 in non‐irradiated vs. 7.17 in irradiated PCs) was not considered substantial. Regarding platelet activation markers, almost no platelet activation was observed. Moreover, platelets retained their aggregability. The proportion of HSRs did not significantly differ between irradiated and non‐irradiated PCs after storage, indicating that UVC‐irradiated platelets maintained their ability to recover from hypoosmotic stress.^23^ Although a higher intensity of UVC irradiation has a greater possibility of causing changes in amino acid residues^24^, ^25^ and may affect platelet quality, the present results suggest that the storage of UVC‐LED‐irradiated platelets for a certain period does not cause substantial damage to their function. The comprehensive evaluation of 265 nm UVC‐LED–irradiated platelets in this study indicated that they were able to withstand storage, which is consistent with previous findings using 254 nm UVC irradiation.^15^

This study has some limitations. First, we used twice the volume of PC as in our previous study—5.5 mL instead of 2.75 mL.^14^ After irradiation, 5 mL of PC was stored in small 15 mL polyolefin bags with high gas permeability. However, in clinical settings, larger PC volumes are used. For example, in Japan, 200–300 mL of PC is stored with gentle agitation in 1000 mL gas‐permeable polyolefin bags or in bags made of polyvinyl chloride. The 15 mL bags used in this study may have been too small to allow for adequate agitation of the 5 mL PC samples, potentially affecting PC quality. Future research should scale up both the irradiation and storage systems to accommodate clinical volumes. Increasing the PC sample volume to 5.5 mL also resulted in a corresponding increase in the sample depth; consequently, a higher fluence was required to achieve bactericidal effects. However, using a shallower sample depth may reduce the effectiveness of UVC irradiation for bacterial inactivation.^8^ These findings highlight the importance of careful system design when developing clinical UVC irradiation platforms.

Second, the reduction in platelet count, whether due to aggregation and/or lysis. Although a small number of aggregates were observed in the PCs, their impact on the in vitro quality of platelets was considered negligible.^25^ The decrease in platelet count was minor and deemed acceptable. Moreover, no further decrease was observed during the subsequent storage. These findings suggest that UVC‐LED‐irradiated PCs may be useful blood components. However, for better quality improvement, further investigations should evaluate the underlying cause of platelet count reduction in such experiments.

In this study, 265 nm UVC‐LED irradiation enhanced the safety of blood transfusions while preserving their efficacy. UVC‐LED irradiation is easy to implement and does not require additional photosensitizers, similar to other UVC‐based PRTs such as those using Hg lamps^8^ and xenon flash light.^26^ Although optimizing the balance between platelet quality and bactericidal efficacy is essential, UVC‐LED treatments are promising methods for improving the safety of platelet transfusions. Further studies are necessary to evaluate the cause of the platelet reduction in our study and the clinical applicability of these findings.

## AUTHOR CONTRIBUTIONS

Tomoya Hayashi performed the experiments, wrote the first draft, and acquired the data; Yoshihiko Sakurai contributed to the writing, editing, and proofreading of the manuscript; Yoshihiro Fujimura designed the study; Tomoya Hayashi and Kumiko Oguma analysed the data; Kumiko Oguma prepared the LED apparatus; Tomoya Hayashi, Yuichi Mishima contributed toward the preparation of blood products; Takafumi Kimura, Yoshihiko Tani, Fumiya Hirayama and Yoshihiro Takihara supervised the study and reviewed and edited the manuscript.

## FUNDING INFORMATION

This study was conducted using research funding from our employer, the Japanese Red Cross Society. The authors received no other specific funding for this study.

## CONFLICT OF INTEREST STATEMENT

Tomoya Hayash, Yoshihiko Sakurai, Yoshihiro Fujimura, Fumiya Hirayama, Yoshihiko Tani, Yoshihiro Takihara and Takafumi Kimura are employees of Japanese red cross society.

## PATIENT CONSENT STATEMENT

All blood donors were given informed consent before using the samples in the study. Parental consent was not obtained because there were no donors young enough to require it.

## Supporting information


**Data S1:** Supporting Information.

## Data Availability

The data that support the findings of this study are available from the corresponding author upon reasonable request.
