# Supplementary material for: Quality of platelet concentrates after three‐day storage following 265 nm ultraviolet C‐light‐emitting diode irradiation
Source: Transfus Med. 2025 Oct 9;36(1):45–52. doi: 10.1111/tme.70025 (PMC12882763; doi:10.1111/tme.70025)
Supplement: Supplementary file 1 — Data S1: Supporting Information. [file TME-36-45-s001.docx]

**Supplementary Information**

**Figure S1 Apparatus for UV irradiation of platelet concentrates**

Irradiation setup is described in the Materials and Methods section.

PTFE, polytetrafluoroethylene; UV, ultraviolet; LED, light-emmiting diode.

**Selection of platelet concentrate** (**PC) storage bags** - Preliminary examination of the quality of PCs after ultraviolet C-light-emitting diodes (UVC-LED) irradiation and storage

**Background**

Considering the small amount of PCs that can be irradiated in the current UVC-LED irradiation system (approximately 5 mL), we selected a blood bag that was suitable for storing small amounts of PC.

**Materials and Methods**

The following six bags were considered.

1. 1000FP (SB-Kawasumi, Kawasaki, Japan; polyolefin resin; 1 000 mL)
2. 80337 (Terumo, Osaka, Japan; polyvinyl chloride; ~1 000 mL)
3. T030 (Terumo; polyvinyl chloride; 300 mL): When storing small amounts of PC, e.g. 40 mL of PC for pediatric cases, T030 is usually used in Japan
4. T008 (Terumo; polyvinyl chloride; 80 mL)
5. 50c (SB-Kawasumi; polyvinyl chloride; 50 mL)
6. Nipro sample culture bag (Nipro; unknown material; 15 mL)

Three days after blood collection, the PC was dispensed in volumes of 2.5 mL to 80 mL depending on the bag size, and stored at 22°C with shaking for three days. We then measured the platelet count, blood gas parameters (pO_2_, pCO_2_), pH, electrolytes (Na+, K+), metabolic parameters (glucose [Glu], lactate [Lac]), and platelet activation markers (PAC-1 binding, CD62P expression, and Annexin V binding), as described in the Materials and Methods section of the main text.

From the results obtained, we first eliminated bags with values that were significantly different from the average, narrowing down the candidates. On the other hand, we tested the correlation between each laboratory test item using the Pearson correlation and Spearman's rank correlation coefficients and identified laboratory tests that correlated highly with other test results. After that, the samples were stored in bags with and without air at 2.5mL and 5.0mL storage volumes, and optimal testing was carried out.

**Results**

The results of the Pearson correlation and Spearman's rank correlation coefficients are shown in Figure S2, indicating that Lac and CD62P levels were strongly correlated with other test items. The T030, T008, 50c, and Nipro sample culture bags, which did not show any outliers in the test results (data not shown), were used in the next examination.

Next, we examined the expression of CD62P after PC storage in the four types of bags that passed the initial screening, and the results indicated that injecting 5.0 mL of PCs without air into the Nipro sample bag was closes to the usual PC storage, 40 mL PC in T030 bag at day 6 (Figure S3).


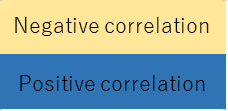

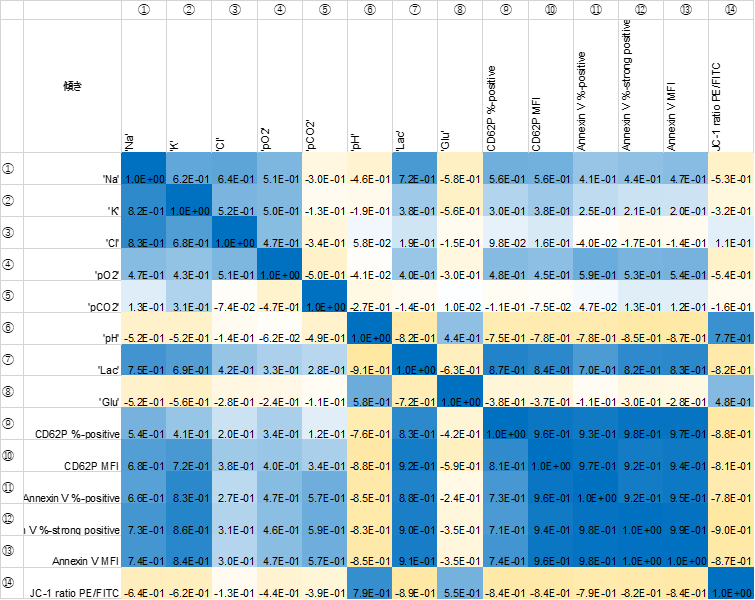

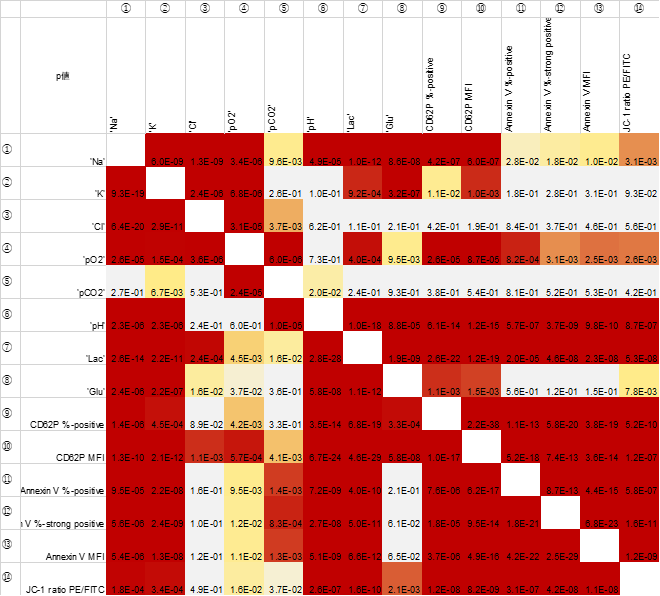


Spearman

Pearson

******Figure S2 Correlation between the results of each test**

**Figure S3 Expression of CD62P after storage in bags with and without air in the bag**

**Platelet activation markers**

**Materials**

The following reagents were used to assess platelet activation markers: Fluorescein isothiocyanate (FITC)-conjugated PAC-1, PerCP-conjugated anti-CD61, and PE-conjugated anti-CD62P antibodies (Becton Dickinson, Tokyo, Japan); FITC-conjugated Annexin V (Sigma-Aldrich, Burlington, MA, USA); protease activator receptor-1 agonist thrombin receptor-activating peptide-6 (TRAP-6; Abcam, Cambridge, UK); and calcium ionophore A23187 (Sigma-Aldrich Japan, Tokyo, Japan). In the analyses of platelet activation markers, platelets used as positive control were diluted to 100 × 10^3^ μL^-1^ with PAS-E before stimulation.

**CD62P surface expression**

The surface expression of CD62P was analysed as previously described.^20^ Platelets activated with TRAP-6 (f.c. 20 μM) for 2 min were used as positive controls for full platelet activation. Platelet aliquots, either immediately or three days post-UVC irradiation, as well as TRAP-6–stimulated platelets—were fixed with cold 1% PFA at 4°C for at least 2 h. After fixation, samples were washed with phosphate buffered saline (PBS) and incubated with PerCP-conjugated anti-CD61 and PE-conjugated anti-human CD62P antibodies for 20 min at room temperature in the dark. PE-conjugated mouse IgG was used as a negative control, instead of the anti-CD62P antibody. After incubation, the samples were washed with PBS and analysed using a SA3800 spectral analyser (Sony, Tokyo, Japan). The proportion of CD62P-positive cells gated to CD61-positive cells was determined.

**PAC-1 binding**

PAC-1 binding was performed according to manufacturer’s instructions and as previously described.^14^ Platelets activated with TRAP-6 (f.c. 20 μM) for 2 min were used as positive controls for full platelet activation. FITC-conjugated PAC-1 and PerCP-conjugated CD61 antibodies were mixed and incubated with platelets either immediately or three days post-UVC irradiation, as well as with TRAP-6–stimulated platelets, for 20 min at room temperature in the dark. After incubation, the platelets were fixed with cold 1% paraformaldehyde (PFA) and stored at 4°C for at least 2 h. After washing with PBS, the samples were analysed using an SA3800 spectral analyser. The median FITC-fluorescence intensity value of cells gated to CD61-positive cells was used to quantify PAC-1 binding.

**Annexin V binding**

Surface PC exposure was assessed using FITC-conjugated Annexin V according to the manufacturer’s instructions. Platelets stimulated with calcium ionophore A23187 (f.c. 10 μM) for 5 min were used as positive controls. Platelet aliquots, either immediately or three days post-UVC irradiation, as well as A23187–stimulated platelets—were diluted in binding buffer (provided in the kit) and incubated with FITC-conjugated Annexin V and PerCP-conjugated anti-CD61 antibodies for 15 min at room temperature in the dark. Samples treated with 10 mM EDTA to block calcium-dependent binding were used as negative controls. After incubation, the samples were fixed with cold 1% PFA at 4°C for at least 2 h, washed with PBS, and analysed using an SA3800 Spectral Analyser. The proportion of FITC-gated CD61-positive cells was determined by Annexin V binding.

**References**

20 Hayashi T, Hayashi A, Fujimura Y, et al. Dual preparation of plasma and platelet concentrates in platelet additive solution from platelet concentrates in plasma using a novel filtration system. *Vox Sang* 2022;117:49-57.

14 Hayashi T, Oguma K, Fujimura Y, et al. UV light-emitting diode (UV-LED) at 265 nm as a potential light source for disinfecting human platelet concentrates. *PLoS One* 2021;16:e0251650.

| Table S1 Correalation between A265 and PLT count | | | | | | | |
| --- | --- | --- | --- | --- | --- | --- | --- |
|  |  |  |  | Fluence | | | |
| sample  number | PLT count | A265 |  | 0 min | 20 min | 40 min | 60 min |
| #2 | 129.3 | 0.591 |  | 0 | 10.5 | 21.0 | 31.6 |
| #3 | 119.5 | 0.535 |  | 0 | 11.6 | 23.2 | 34.9 |
| #4 | 165.4 | 0.645 |  | 0 | 9.6 | 19.3 | 28.9 |
| #5 | 151.9 | 0.579 |  | 0 | 10.7 | 21.5 | 32.2 |
| #6 | 91.8 | 0.522 |  | 0 | 11.9 | 23.8 | 35.7 |
| #7 | 95.8 | 0.536 |  | 0 | 11.6 | 23.2 | 34.8 |
| #8 | 88.5 | 0.59 |  | 0 | 10.5 | 21.1 | 31.6 |
| #9 | 140.1 | 0.608 |  | 0 | 10.2 | 20.5 | 30.7 |
| minimumn | 88.5 | 0.522 |  | 0.0 | 9.6 | 19.3 | 28.9 |
| maximumn | 165.4 | 0.645 |  | 0.0 | 11.9 | 23.8 | 35.7 |
| Mean | 122.8 | 0.576 |  | 0.0 | 10.8 | 21.7 | 32.5 |
| SD | 29.0 | 0.042 |  | 0.0 | 0.8 | 1.6 | 2.4 |

PLT, platelet

**Figure S4** Correalation between A265 and PLT count

D)

C)

B)

A)

**Figure S5 Irradiation with 265 nm UVC-LED reduced bacterial colony formation in platelet concentrate (PC)**

PCs inoculated with *Staphylococcus aureus* (A, C) or *Bacillus cereus* (B, D) were irradiated. (A, B) Ratio of the colony counts to that at 0 min of irradiation. Closed circle: with irradiation; open circle: without irradiation (control). The mean colony counts were 54 (*S. aureus*, irradiated), 55 (S. aureus, not irradiated), 23 (B. cereus, irradiated), and 27 (*B. cereus*, not irradiated). (C, D) The mean fluence was calculated (n = 6), and the log of the reduction ratio was plotted. The CFU count reflecting low detection was set to log survival = −2. The mean fluences for S. aureus and B. cereus, respectively, were 13.5 and 13.0 mJ cm-^2^ (after 20 min irradiation); 27.0 and 25.9 mJ cm^-2^ (after 40 min irradiation); and 40.4 and 38.7 mJ cm-^2^ (after 60 min irradiation) (C, D). The slopes of the regression lines are -0.0445 (C) and -0.0301 (D). The slope units were calculated as (log reduction)/(mJ cm-^2^). CFU, colony-forming unit; UVC-LED, ultraviolet C-light-emitting diodes
